# NOT "Good Enough for the War."

**Published:** 1916-08-19

**Authors:** 


### THE MATRONS' AND SISTERS' DEPARTMENT.

### NOT "GOOD ENOUGH FOR THE WAR."

Before the war Great Britain had amongst its voluntary hospitals the best and most perfect types of the modern hospital in the world. This excellence in administration was often due to ability and devotion on the part of one or more of the higher officials, but its vitality and force resulted from the high standards of efficiency maintained throughout the institution. A state of war, and such a war as the present one, which was forced upon the nation all unprepared, and forced insistently, necessarily created huge difficulties at the outset, not the least of which was to find someone everywhere to be doing something in the way of a start. This fact necessitated a blind eye on the part of those who were aiming at improvement and higher efficiency in the first year of the war. We are now, however, in the third year of war, and time and circumstances have demonstrated where there are leaks in those portions of the hospital system which are directly associated with the fighting forces. Inefficiency with an absence of standard is necessarily associated with the great whitewasher, who has become notorious since the commencement of the war for glossing over and hushing up defective administration and jobs of various kinds. Fortunately many of the larger givers are alive to the evils attaching to the influence of persons of the great whitewasher type, and its high priest may mercifully yet be thrust aside and rendered impotent for further mischief in the future.

One lamentable weakness brought out by the war in this connection has been the retention in high office of individuals after their unfitness has been demonstrated beyond dispute. Owing their position possibly to private influence, once appointed the defects and failures which have resulted have been tolerated and their services retained on the plea that it was only for the war, and the war will soon be over. Anything more mischievous or wrong in the administrative sense, having regard to the interests of an enormous staff of workers and of an infinite and ever-changing number of sick and wounded warriors of both Services, it would be impossible to imagine. We have not failed to speak out on more than one occasion when marked incompetence has been exhibited by the presence of inefficiencies in a great department or an important war hospital. In both fields some changes are urgently called for. Having regard to the fact that no one can say how long the war will endure, it is surely time to survey the holders of high office throughout the nursing departments of the Army and Auxiliary Forces with a view to make certain changes which have long been called for. "Good enough for the war," like that discredited utterance "Wait and see," has had its day, and must be dropped by the substitution of a higher standard of administrative control at the centre.

These thoughts have been given substance by the semi-official explanation which has been offered for the inhygienic and dangerous conditions under which the nursing staff at one hospital have to live, and many of its individual members have been given sleeping quarters that are unfit for the habitual use of trained nurses of any rank who have to keep smartly efficient and continuously in attendance upon the wounded and the sick. We make bold to say that no such condition of affairs could possibly arise, much less could have continued, in any hospital where the head of the nursing staff is possessed of energy, high competence, and true ideals of her duty and responsibility. A remonstrance was sent us from the centre that our criticisms in our issue of the 5th instant on the nurses' sleeping quarters at the King George Hospital were too severe. The present quarters were not so bad, and were "good enough for the war," which we seemed to have forgotten. We had further overlooked the fact that it was only the nurses whose beds were placed on the side of the wall without windows or ventilation who complained, and that those nearer the door had quite as much air as they needed. The telephone's is not unfortunately a written communication, but a telephone defence such as that just given is amazing testimony to the fact that the plainest statement of remonstrance is imperative on the part of those who are responsible for leading public and nursing opinion in these matters. Our protest in our issue of the 5th instant was deliberate and purposeful. It ought to have produced an immediate change, and that it would have done so in 99 per cent. of the hospitals in these islands we can testify from experience. Either the administration of King George Hospital will have to be changed and reorganised, so far as its nursing department is concerned, or its present name should no longer be associated with this institution.

We have received a letter of thanks and other letters, and have written evidence of the need for reform and the miseries and risks to the nurses and women employees. There is no proper organisation of the food of the night nurses, and it is monstrous to keep them without ample food and a hot meal under suitable conditions. The organisation of the work in the wards from the very outset to the present time has been defective, and could not have occurred had there been efficient supervision resting upon knowledge and a high standard of responsibility. We have no space to go further into the matter on the present occasion, but we wish to say as plainly as words can speak that these matters should be mended, or they and those responsible for them should be ended without further delay. "Good enough for the war" should surely find its quietus in the presence of the defective sleeping quarters and current abuses at the King George Hospital.

